# Second harmonic generation from the ‘centrosymmetric’ crystals

**DOI:** 10.1107/S2052252515002183

**Published:** 2015-03-20

**Authors:** Venkatram Nalla, Raghavender Medishetty, Yue Wang, Zhaozhi Bai, Handong Sun, Ji. Wei, Jagadese J. Vittal

**Affiliations:** a Centre for Disruptive Photonic Technologies, Nanyang Technological University, Singapore 637371, Singapore; b Department of Chemistry, National University of Singapore, 3 Science Drive 3, Singapore 117543, Singapore; c Division of Physics and Applied Physics, School of Physical and Mathematical Sciences, Nanyang Technological University, Singapore 637371, Singapore; d Department of Physics, National University of Singapore, 3 Science Drive 3, Singapore 117542, Singapore

**Keywords:** second harmonic generation, centrosymmetric crystals

## Abstract

Second harmonic generation (SHG) is a well known non-linear optical phenomena which can be observed only in non-centrosymmetric crystals due to non-zero hyperpolarizability. In the current work we observed SHG from a Zn(II) complex which was originally thought to have crystallized in the centrosymmetric space group *C*2/*c*. This has been attributed to the unequal antiparallel packing of the metal complexes in the non-symmetric space group *Cc* or residual non-centrosymmetry in *C*2/*c* giving rise to polarizability leading to strong SHG. The enhancement of SHG by UV light has been attributed to the increase in non-centrosymmetry and hence polarity of packing due to strain induced in the crystals. The SHG signals measured from these crystals were as large as potassium dihydrogen phosphate crystals, KH_2_PO_4_ (KDP), and showed temperature dependence. The highest SHG efficiency was observed at 50 K. The SHG phenomenon was observed at broad wavelengths ranging from visible to below-red in these crystals.

## Abstract 2

Based on the observation of second harmonic generation (SHG), the solid state structure of a Zn(II) complex has been reinterpreted in terms of the non-centrosymmetric space group *Cc*, with residual polarity arising from unequal antiparallel packing. Temperature-dependent measurements showed that the highest SHG has been observed at 50 K and the lowest at 170 K.

## 1. Introduction   

One of the most ubiquitous problems encountered in the crystal structure analysis of non-chiral molecules is the ambiguity between the centrosymmetric and non-centrosymmetric space groups such as *P*1 – 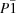, *P*2_1_ – *P*2_1_/*m*, *C*2 – *C*2/*m*, *Cc* – *C*2/*c*, *Pna*2_1_ – *Pnma*, among others (Marsh, 1986 ▶, 1994 ▶; Dougherty & Kurtz, 1976 ▶). In other words, a crystal structure can contain centrosymmetric packing (with inversion symmetry) of molecules exclusively or an equal amount of enantiomers, *i.e.* cancellation of polarity in packing in non-centrosymmetric space groups. Due to these uncertainties between the space groups the description of structures in centrosymmetric packing has been advocated, in the absence of supportive evidence for non-centrosymmetric space groups (Baur & Tillmanns, 1986 ▶). Modern crystallographic software available nowadays can clearly help in resolving these space-group ambiguities, in addition to parameters like Flack, Parsons and Hooft (Schomaker & Marsh, 1979 ▶; Marsh & Herbstein, 1983 ▶; Flack, 1983 ▶; Hooft *et al.*, 2008 ▶; Parsons & Flack, 2004 ▶). On the other hand, the absence of inversion symmetry is one of the important requirements for properties such as pyroelectricity, piezoelectricity, ferroelectricity, triboluminescence and second harmonic generation (West, 1999 ▶; Smart, 2012 ▶). Even a small ‘polarity’ excess in the mixture of non-centrosymmetric crystals formed during synthesis can exhibit some of these interesting solid-state properties mentioned above (Molinos-Gómez *et al.*, 2007 ▶; Jaya Prakash *et al.*, 2008 ▶; Shakir *et al.*, 2009 ▶; Clevers *et al.*, 2013 ▶; Mishuk *et al.*, 2014 ▶).

Second harmonic generation (SHG) is a nonlinear optical phenomenon related to nonlinear electrical susceptibility χ^(2)^. For centrosymmetric crystals, based on the Kleinman approximation symmetry and centrosymmetry of crystals, χ^(2)^ has no independent irreducible component of tensors, thus exhibiting no SHG effect (Lee & Kim, 2012 ▶). As a result, non-centrosymmetric packing is a prerequisite for the observation of SHG (Zyss & Oudar, 1982 ▶). In recent years, the SHG effect from centrosymmetric crystals with vicinal faces has been reported (Verheijen *et al.*, 1991 ▶). Considering the SHG arising from bulk electric quadrupole and magnetic dipole effects and a surface dipole source, Lüpke presented a phenomenological theory (Lüpke *et al.*, 1994 ▶; Sipe *et al.*, 1987 ▶) to account for the observed SHG properties. In the last few years there was a resurgence of interest in SHG from cubic centrosymmetric crystals (Guo *et al.*, 2002 ▶; Cazzanelli *et al.*, 2012 ▶). SHG measurements could be utilized to probe non-centrosymmetric surface structures which are clearly distinguishable from the bulk structure (Sipe *et al.*, 1987 ▶). Furthermore, SHG exhibited by such single crystals has been scrutinized before (Kurtz, 1968 ▶).

Recently, we investigated the photosalient properties of centrosymmetric crystals of three Zn(II) complexes under UV light (Medishetty *et al.*, 2014 ▶). To our surprise, only one of these crystals exhibited SHG properties that were comparable with KDP, a well known crystal capable of generating SHG signals efficiently. Hence we have investigated the origin of SHG properties in this compound, and proposed that the presence of a slight polarity excess in the otherwise antiparallel packing in the space group *C*2/*c* and the enhancement of surface defects by UV irradiation should be responsible for its observed behavior. The details of our investigations are presented and discussed below.

## 2. Results and discussion   

### 2.1. Crystal structure description   

**Figure 1:**
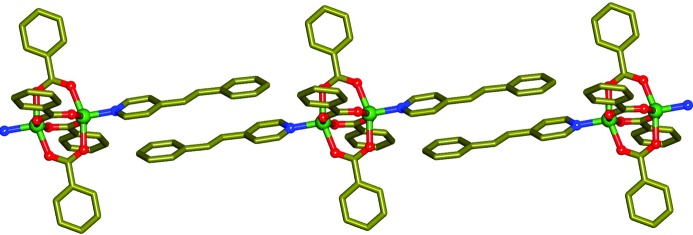
Ball and stick diagram of **1**. Color coding: Zn – green, C – yellow, O – red, N – blue.

During the slow evaporation of a methanolic solution of Zn(NO_3_)_2_, sodium benzoate and 4-styrylpyridine (4spy) in a 1:2:1 molar ratio, the coordination metal complex [Zn_2_(benzo­ate)_4_(4spy)_2_] (**1**), was obtained (Fig. 1 ▶). Single-crystal X-ray diffraction (XRD) revealed that the compound crystallizes in *C*2/*c* with *Z* = 4 (CCDC No. 979138). The asymmetric unit consists of half of the molecular formula where Zn(II) is present in square-pyramidal geometry and these Zn(II) atoms are bridged by four benzoate groups leading to the formation of a well known paddle wheel building unit. The axial sites of these units are coordinated by the N atoms of the 4spy ligand (Medishetty *et al.*, 2014 ▶). This coordinated 4spy interacted with another 4spy in a *head-to-tail* fashion with π–π interactions between pyridine and the phenyl group, forming a supramolecular one-dimensional polymer. A crystallographic inversion centre is present in the middle of the paddle-wheel structure with half the formula units present in the asymmetric unit. Variable-temperature single-crystal X-ray data in conjunction with differential scanning calorimetry (DSC) studies indicate that there is no phase change observed in the temperature range 223–373 K.

Although the compound is crystallized in the centrosymmetric space group, to our surprise these crystals exhibit a second-order non-linear optical property as confirmed from strong SHG signals measured using 6 ns laser pulses of wavelengths ranging from 850 to 1200 nm. The details of our investigations are described below.

### 2.2. Laser input energy-dependent SHG spectra of **1** and KDP   

**Figure 2:**
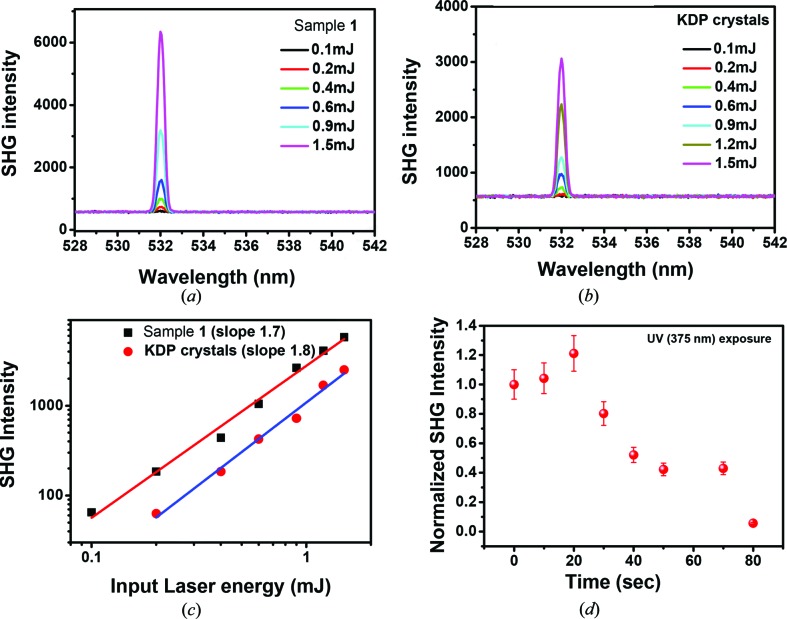
Laser input energy-dependent SHG spectra of (*a*) single crystals of **1**, (*b*) KDP single crystals, (*c*) laser input energy-dependent SHG intensity of KDP and **1**, and (*d*) UV exposure time dependence of SHG of **1**.

Freshly synthesized single crystals of **1** dried at room temperature were exposed to 850–1200 nm and 6 ns laser pulses on a glass slide with a microscope (Figs. S2 and S3). The signal was coupled to a monochromator and the CCD, emission wavelength and intensity were measured using a CCD detector (Fig. S2). Using the same set-up, the SHG emissions of KDP were also measured as a reference. Input laser energy-dependent SHG spectra from KDP and **1** are shown in Figs. 2 ▶(*a*) and (*b*). Input laser energy-dependent SHG intensities of the crystals of KDP and **1** are shown in Fig. 2 ▶(*c*), which clearly shows that the SHG intensity in **1** is two times higher than KDP. Slopes of the laser energy dependence are 1.8 and 1.7 for the crystals of KDP and **1**, respectively. The scattered light on a metal mirror and the glass slide were also measured showing a negligible dark signal at 532 nm (*i.e.* the corresponding SHG signal). These results support the fact that the emission is due to its non-linearity of **1**. This has been confirmed from several crystals of **1** from several batches of crystallization. Although the absolute intensity of the SHG signals vary among crystals, the reproducibility of SHG behaviour in **1** has been demonstrated unequivocally. These crystals can also generate SHG in a range of near below-red wavelengths (Fig. S5). During the SHG experiment, when the freshly prepared crystals were irradiated under UV light, the intensity of the SHG increased and slowly faded away on continued exposure to UV light as shown in Fig. 2 ▶(*d*). The increase in intensity could be attributed to the strain created by UV irradiation (Cazzanelli *et al.*, 2012 ▶), and the decrease in intensity could be due to the formation of a new photodimerized product (Medishetty *et al.*, 2014 ▶).

### 2.3. Temperature-dependent SHG   

**Figure 3:**
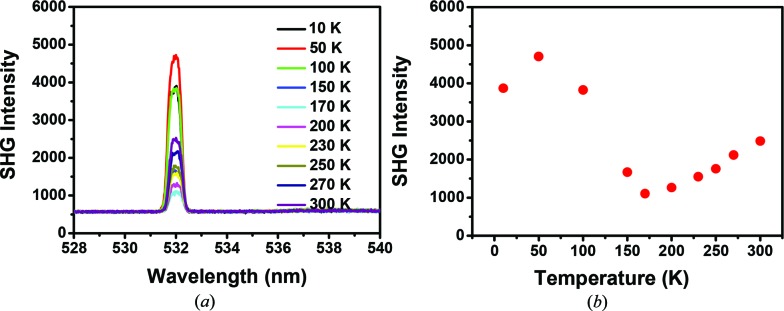
(*a*) Temperature-dependent SHG spectra of **1**. (*b*) Temperature-dependent SHG intensity of **1** excited at 1064 nm wavelength, 20 Hz repetition rate, 1.5 mJ energy pulse and 6 ns pulse width.

Temperature-dependent SHG measurements have been carried out in the temperature range 10–300 K. As shown in Fig. 3 ▶, a freshly prepared sample was placed in a small glass cup and loaded in a cryostat. The SHG signals were measured at different temperatures by slowly increasing to room temperature from 10 K and keeping the input laser energy at 1.5 mJ. These curves clearly demonstrate that the SHG intensity varies with temperature to a maximum at ∼ 50 K and then gradually decreases with an increase in temperature until 170 K, and finally increases again from 170 K to room temperature. This temperature-dependent measurement indicates that the maximum SHG intensity is observed at 50 K and the minimum at 170 K (Fig. 3 ▶ *b*). This observation is similar to previous reports for many non-centrosymmetric crystals (Lee & Kim, 2012 ▶).

Although the SHG effect has been found to enhance due to the strain created by exposure to UV light, it was still not clear why a centrosymmetric crystal shows this nonlinear optical (NLO) property in the first place. A number of so-called ‘centrosymmetric crystals’ have been found to exhibit the SHG effect before (Shakir *et al.*, 2009 ▶; Guo *et al.*, 2002 ▶). Desiraju *et al.* (1979 ▶) found that the crystal structures of the space group *P*2_1_/*c* were indeed a mixture of two non-centrosymmetric space groups, namely *P*2_1_ and *Pc*. As early as 1935, Robertson scrutinized complexes with phthalocyanines and metals for non-centrosymmetric packing (Robertson, 1935 ▶). In order to trace the origin of NLO behaviour, crystals of **1** synthesized in the dark were carefully tested for their SHG and were found to show SHG effects irrespective of UV light exposure. Hence, it was concluded that this may be an inherent property of the crystals. Secondly, it was thought that the non-centrosymmetry may be lost due to highly intense X-rays during the data collection. Hence the same crystals show that NLO behaviour was used for single-crystal data collection, and tested again for their SHG properties. It has been found that there is no change in the SHG intensity confirming that there is no change in symmetry of the crystals due to X-ray exposure. Then why do the centrosymmetric crystals show very high SHG intensity? Could this be due to the residual polarity in the packing of the almost antiparallel packing of the metal complexes in *Cc* or local non-centrosymmetry in the otherwise bulk centrosymmetric crystals responsible for this observed property? If this is true, the emission intensity is expected to vary, at least slightly, with crystals obtained in the same or different batches of crystallization. To confirm this hypothesis, a number of single crystals obtained from the same batch of synthesis were examined under laser excitation. The intensities of the SHG were indeed found to vary in these batches, and thus lend support for the presence of local non-centrosymmetry in the otherwise bulk centrosymmetric packing of molecules in *C*2/*c* present in the single crystals. Actually, when the crystal structure refinements were carried out in *Cc*, the absolute structure parameter (Flack parameter) was refined close to 0.5 for the data in the range 90–298 K for various single crystals. Further, we have collected data at different temperatures and crystals showing different SHG intensities (Fig. 2 ▶ *d*, Fig. S6). However, we were unable to find any direct correlation between the SHG intensities measured and the Flack or other parameters. Furthermore, [Zn_2_(benzoate)_4_(2F-4spy)_2_] (**2**, where 2F-4spy = 2-fluorophenyl-4-strylpyridine) and [Zn_2_(benzoate)_4_(3F-4spy)_2_] (**3**, where 3F-4spy = 3-fluorophenyl-4-strylpyridine) were also found to be isomorphous and isostructural with **1** and exhibit photosalient properties similar to **1**. On the contrary, these two crystals do not show any SHG properties, suggesting that they belong to the centrosymmetric space group *C*2/*c* unlike **1**. Finally, it is worth mentioning that the SHG signal of **1** disappeared after grinding the single crystals into powder. Besides these, it was also found that **1** grown from ethanol has different morphology and did not show any SHG signal which implies that they belong to the centrosymmetric crystals like **2** and **3** (Medishetty *et al.*, 2014 ▶).

In summary, this communication reports so-called ‘centrosymmetric’ crystals of a Zn(II) complex, which is indicated surprisingly to have crystallized with a residual non-centrosymmetry in space group *C*2/*c* based on the observation of SHG. A small excess of polarity packing in the otherwise centrosymmetric single crystals appears to result in a large second-order nonlinearity which can be further enhanced by increasing the strain caused by the UV light exposure. These led to a strong dependence of χ^(2)^ on the inhomogeneity created by the induced strain. The induced strain varies with temperature, as indicated from the temperature-dependent SHG measurements. By increasing the temperature from 10 to 50 K, a sharp increase in the SHG signal is followed by a slow decrease to its minimum at ∼ 175 K. A further increase in the temperature to 300 K is accompanied by a slow increase in the SHG signal. The underlying mechanism has been discussed.

## 3. Experimental   

### 3.1. Materials and general methods   

Commercially available reagent-grade chemicals were used as received without any further purification unless mentioned. IR spectra were recorded on a FTS165 Bio-Rad FTIR spectrometer by using KBr pellets in the range 4000–400 cm^−1^. Elemental analysis (C, H and N) was carried out using an Elementar Vario Micro Cube instrument at the Elemental Analysis Lab, CMMAC, Department of Chemistry, National University of Singapore. Thermogravimetric analysis (TGA) was performed under N_2_ atmosphere with a heating rate of 5°C min^−1^ on a SDT 2960 Thermal analyzer. NMR spectra were recorded on a 300 MHz Bruker Avance 300 FT-NMR spectrometer by calibrating the residual solvent as the reference in DMSO-d_6_ solution. The powder X-ray diffraction (PXRD) patterns were recorded on a Siemens D5005 diffractometer with graphite monochromated Cu *K*α radiation (λ = 1.54056 Å) at 298 K.

### 3.2. SHG measurements   

SHG by the MOC crystals was measured with 6 ns laser pulses (850–1200 nm wavelength and 20 Hz repetition rate) emitted from a Q-switched Nd:YAG (yttrium-aluminium-garnet) laser (Spectra Physics, INDI) pumped OPO. The laser pulses were focused onto the sample on a glass plate with a spot size of 200 μm. The sample was kept in a cryostat whose temperature was varied for the measurement of temperature-dependent SHG signals. Optical density filters were used to change incident laser energy. The signal was collected using two collimation lenses and dispersed by a 750 mm monochromator (Hamamatsu R928) and the spectrum was recorded by a charged coupled device (CCD, Princeton Instruments). During the measurement, a dichroic filter was used to block the pump beam. A UV lamp (375 nm) was used for UV enhanced SHG generation studies.

### 3.3. Synthesis of [Zn_2_(benzoate)_4_(4spy)_2_] (1)   

Colourless rod-like single crystals were obtained from slow evaporation of a methanol solution of Zn(NO_3_)_2_·6H_2_0 (15.0 mg, 0.05 mmol), the sodium salt of benzoic acid (14.4 mg, 0.1 mmol) and 4spy (9.1 mg, 0.05 mmol) and dried at room temperature. Yield: 23.1 mg (60%). The elemental analysis (%): calculated for C_27_H_21_NO_4_Zn: C 66.33, H 4.33, N 2.87; found: C 65.94, H 3.91, N 2.83. ^1^H NMR (DMSO-d_6_, 300 MHz, 298 K): δ = 8.58 (d, 4H, pyridyl protons of 4spy), 7.2–7.7 (m, 18H, aromatic protons of 4spy), 7.96 (d, 8H, benzoate protons), 7.2–7.5 (m, 12H, benzoate protons). IR (KBr pellet, cm^−1^): 1637, 1574, 1505, 1401, 1226, 1172, 1069, 1032, 965, 873, 839, 816, 715, 690, 538, 458.

### 3.4. X-ray crystallography   

Structural data for all these crystals were collected on a Bruker APEX II diffractometer attached with a CCD detector and graphite-monochromated Mo *K*α (λ = 0.71073 Å) radiation through a sealed tube (2.4 kW). An empirical absorption correction was applied to the data using the *SADABS* (Sheldrick, 1996 ▶) program and the crystallographic package *SHELXTL* (Sheldrick, 2008 ▶; Müller *et al.*, 2006 ▶) was used for all calculations. The crystal data were refined purposely in the space group *Cc* to show the presence of both non-centrosymmetric and centrosymmetric packing in the crystal. The crystal was originally refined in the space group *C*2/*c* before (Medishetty *et al.*, 2014 ▶, CCDC No. 979138). The crystallographic data for CCDC 1031432–1031436 can be found in the supporting information and from the Cambridge Crystallographic Data Centre *via* http://www.ccdc.cam.ac.uk/data_request/cif.

#### 3.4.1. Crystal data for MOC crystals at 170 (2) K   

**1** at 170 (2) K: C_54_H_42_N_2_O_8_Zn_2_, *M*_r_ = 977.64, monoclinic, *Cc*; *a* = 24.746 (2), *b* = 12.212 (1), *c* = 15.653 (1) Å, β = 109.192 (1)°, *V* = 4467.2 (6) Å^3^, *Z* = 4, ρ_calc_ = 1.454 g cm^−3^, μ = 1.134 mm^−1^, GOF = 1.046, final *R*1 = 0.0323, *wR*2 = 0.0845 [for 8188 data *I* > 2σ(*I*)].

## Supplementary Material

Crystal structure: contains datablock(s) global, e210, e219, e257, e254, e251. DOI: 10.1107/S2052252515002183/ed5004sup1.cif

Structure factors: contains datablock(s) E210-RT. DOI: 10.1107/S2052252515002183/ed5004e210sup2.fcf

Structure factors: contains datablock(s) e219. DOI: 10.1107/S2052252515002183/ed5004e219sup3.fcf

Structure factors: contains datablock(s) e257. DOI: 10.1107/S2052252515002183/ed5004e257sup4.fcf

Structure factors: contains datablock(s) e254. DOI: 10.1107/S2052252515002183/ed5004e254sup5.fcf

Structure factors: contains datablock(s) e251. DOI: 10.1107/S2052252515002183/ed5004e251sup6.fcf

Extra tables and figures. DOI: 10.1107/S2052252515002183/ed5004sup7.pdf

CCDC references: 1031432, 1031433, 1031436, 1031435, 1031434
