# Supplementary material for: Second harmonic generation from the ‘centrosymmetric’ crystals
Source: IUCrJ. 2015 Mar 20;2(Pt 3):317–21. doi: 10.1107/S2052252515002183 (PMC4420541; doi:10.1107/S2052252515002183)
Supplement: Supplementary file 7 [file m-02-00317-sup7.pdf]

IUCrJ

Volume 2 (2015)

Supporting information for article:

Second harmonic generation from the "centrosymmetric" crystals

Venkatram Nalla, Raghavender Medishetty, Wang Yue, Zhaozhi Bai, Nikolay I. Zheludev, Sun Handong, Ji Wei and Jagadese J. Vittal

Second harmonic generation from the “centrosymmetric” crystals

Venkatram Nalla[a], Raghavender Medishetty[b], Wang Yue[c], Zhaozhi Bai[b], Handong Sun[a*], Wei Ji[d]* and Jagadese J. Vittal[b]*

[a]Centre for Disruptive Photonic Technologies, Nanyang Technological University, Singapore, 637371
[b]Department of Chemistry, National University of Singapore, 3 Science Drive 3, Singapore 117543
[c]Division of Physics & Applied Physics, School of Physical & Mathematical Sciences, Nanyang Technological University, Singapore 637371
[d]Department of Physics, National University of Singapore, 3 Science Drive 3, Singapore, Singapore 117542

Supporting information

The crystal data were refined purposely in the space group *Cc* to show the presence of both non-centrosymmetric and centrosymmetric packing in the crystal. The crystal was originally refined in the space group *C*2/*c* before (Medishetty, 2014, CCDC No. 979138).

Table S1 Cell data for **1** at different temperatures

	**1 (E219)**	**1 (E210)**
Temperature (K)	*170(2)*	*295(2)*
Sp. Grp	*Cc*	*Cc*
***a* (Å)**	24.746(2)	24.843(2)
***b* (Å)**	12.212(1)	12.245(1)
***c* (Å)**	15.653(1)	15.720(1)
**β (deg)**	109.192(1)	109.498(2)
**V (Å^3)**	4467.2(6)	4507.9(7)
Z	4	4
**D_{calc}(g/cm^3)**	1.454	1.440
**μ (mm^{-1})**	1.134	1.124
**GOF**	1.046	1.010
**R_1 ($I > 2\sigma(I)$)**	0.0323	0.0371
**wR_2 ($I > 2\sigma(I)$)**	0.0845	0.0898

Only selected data are shown here since they are very similar and no trend has been found. Other data can be retrieved: CCDC 1031432-1031436

IMPORTANT: this document contains embedded data - to preserve data integrity, please ensure where possible that the IUCr Word tools (available from http://journals.iucr.org/services/docxtemplate/) are installed when editing this document.

Table S2 The Flack parameters for **1** obtained for two single crystals at different temperatures.

Temperature	SHG found	Flack parameter for the Crystal showing High SHG	Flack parameter for the Crystal showing Low SHG
170 K	low	0.34(3)	0.38(3)
295 K	Medium	0.10(3)	0.41(3)
80 K	high	-	0.37(3)

The Flack and Hooft parameters were also obtained using OLEX2 but the parameters changed and no correlation was found.

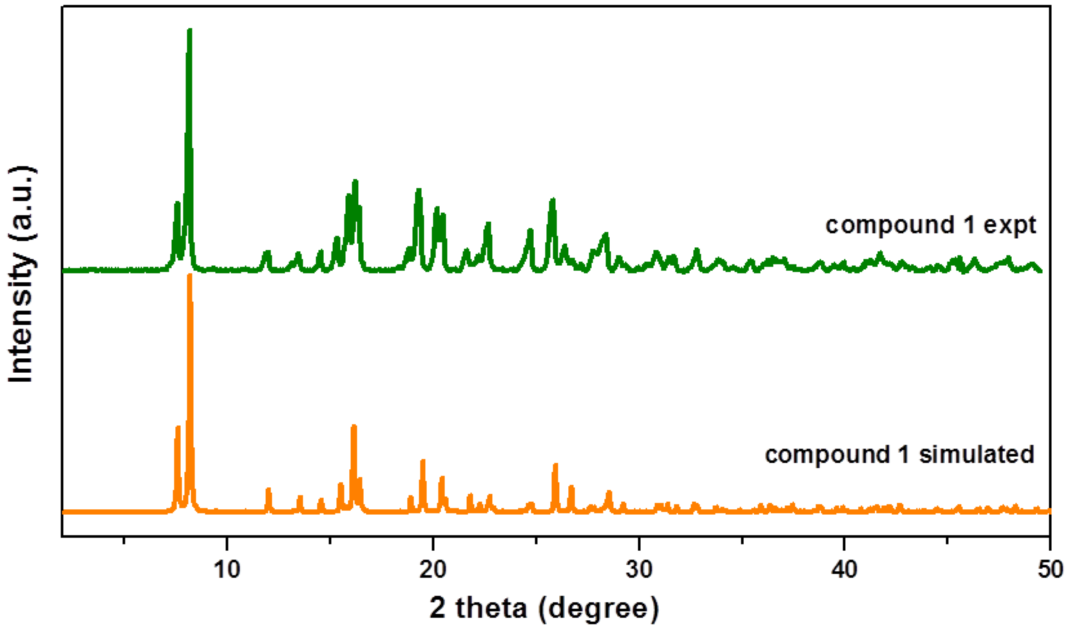

Figure S1 PXRD pattern of **1**.

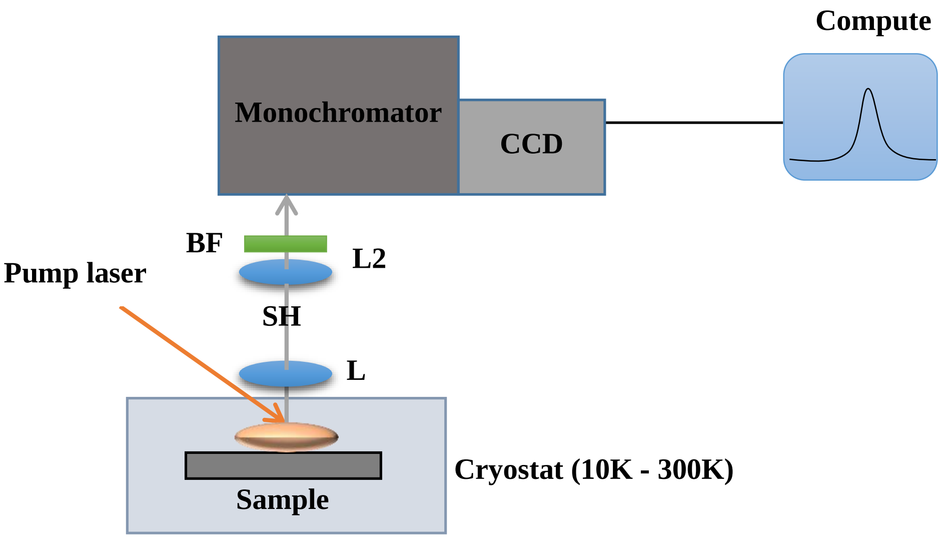

Figure S2 Schematic view of SHG measurements.

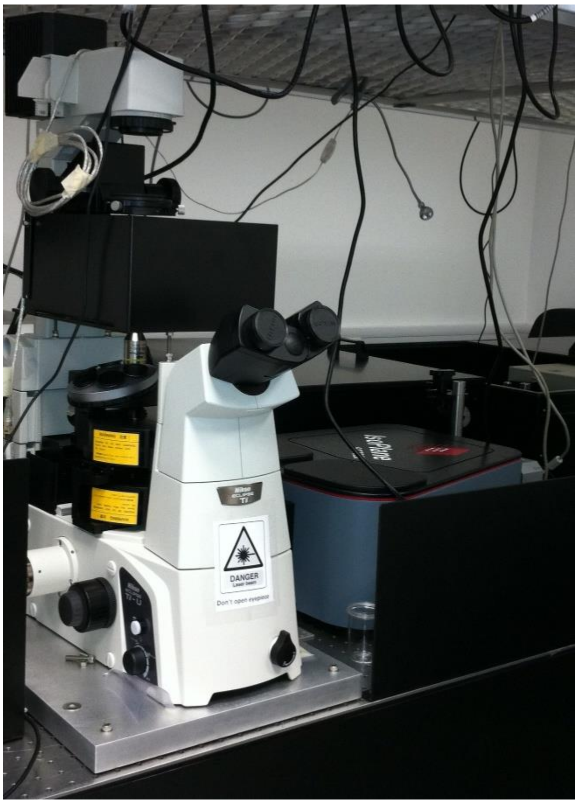

Figure S3 Experimental microscope set up for SHG measurement at room temperature.

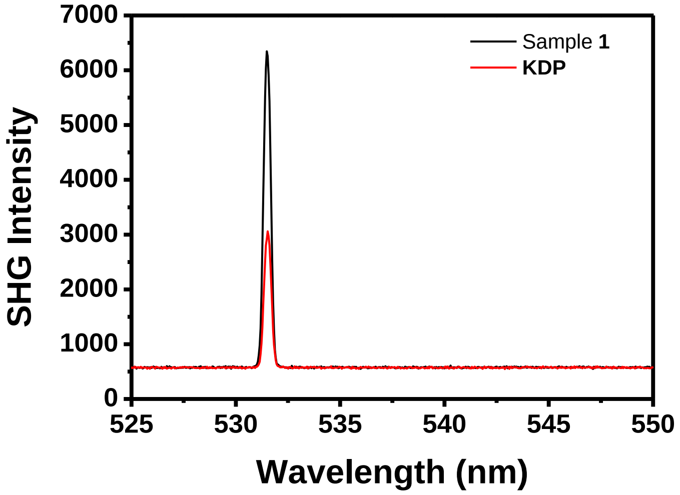

Figure S4 SHG measurement of KDP and **1**.

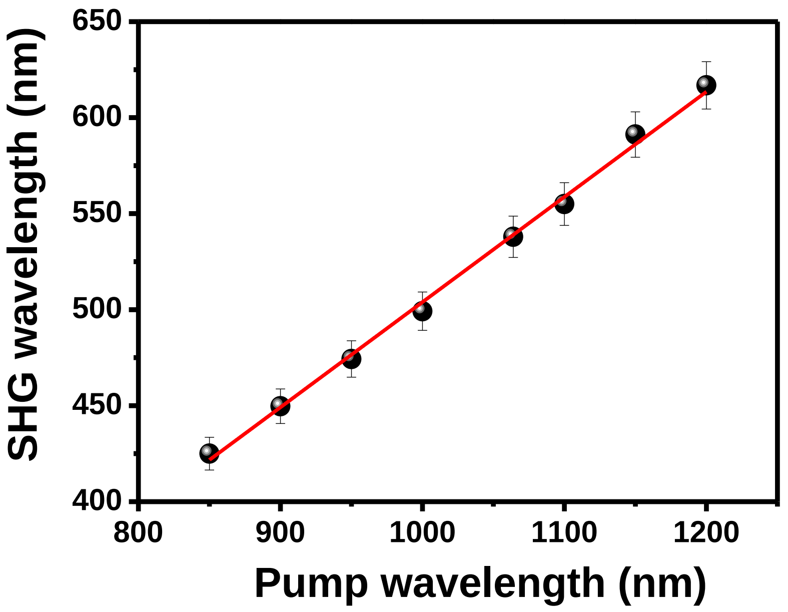

Figure S5 Excitation wavelength dependent SHG wavelength measurement of **1**.

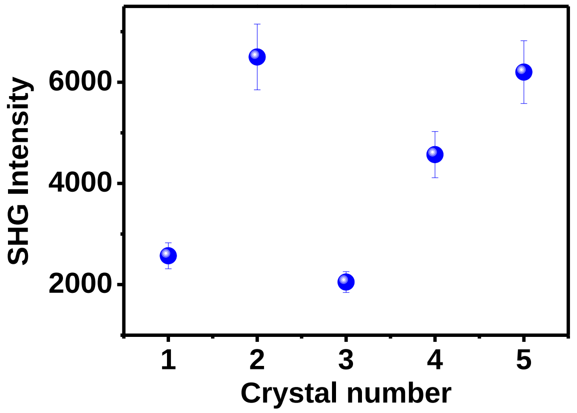

Figure S6 SHG intensities of different crystals from the same batch of **1**.
